# Brazil’s experiment to expand its medical workforce through private and public schools: Impacts and consequences of the balance of regulatory and market forces in resource-scarce settings

**DOI:** 10.1186/s12992-025-01105-8

**Published:** 2025-03-28

**Authors:** Mário Scheffer, Paola Mosquera, Alex Cassenote, Barbara McPake, Giuliano Russo

**Affiliations:** 1 Preventative Medicine Department, Faculty of Medicine, University of São Paulo, Avenida Dr. Arnaldo, 455, 2º andar, sala 2166, São Paulo, CEP: 01246-903 Brazil; 2 Melbourne School of Population and Global Health, The University of Melbourne, Level 4, 207-221 Bouverie St, Melbourne, VIC 3010 Australia; 3 The Wolfson Institute of Population Health, Queen Mary University of London, 58 Turner Street, London, E1 2AB UK

**Keywords:** Physicians, Medical schools, Scaling-up policies, Health workforces, Health markets in LMICs

## Abstract

**Background:**

There is a global shortage of doctors, and governments worldwide are concerned with expanding national medical workforces to improve services. Since 2013 the Government of Brazil has introduced the *Mais Médicos* (More Doctors) Legislation (MML), which included policies to liberalise the medical education market and boost deployment to rural areas, and implemented quotas in public universities to improve diversity in the supply of physicians. Such experience provides an insight for the global debate on the role of the private sector in medical education.

**Methods:**

We draw from the analysis of unique medical demography datasets to assess the impact of those policies on the number and distribution of doctors and medical students, composition of the workforce, and quality of training. To analyze the increasing trend of students and physicians, interrupted time-series analysis was conducted using segmented linear regression, comparing two time periods considering the MML as the start of the intervention. Staff-to- student ratios and ENADE educational attainment data were used to compare the quality of teaching between public and private institutions.

**Findings:**

Within the context of Brazil’s population and economic growth over the last decades, we find that since 2003 Brazil has almost doubled its medical workforce to 2.77 per 1,000 population, with the largest increase recorded after the 2013 legislation. Our analysis shows such growth has benefited poorer, remote states, although the bulk of new doctors and students are still located in the country’s richer regions. The diversity of medical students increased significantly since the More Doctors Legislation, with more female (61.4% in 2023 as opposed to 55.5% in 2013), and mixed-race enrolments (25.5% and 19.4%). However, medical students are still predominantly white (68.7% and 71.6%), and from fee-paying secondary schools (68.1% and 75.8%). Comparison of student achievement scores and of deployed resources also show a significantly lower quality of teaching in private medical schools.

**Conclusions:**

We conclude that Brazil’s policy approach has delivered a substantial overhaul of its medical workforce through a combination of public and private sector policies. However, progress in students’ diversity and quality of education has been mixed. Brazil’s experiment suggests that private schools can be an option for rapid health workforce expansions in middle-income economies. However, close monitoring of their outputs would be needed, as our analysis shows they do little to address inequalities, and casts doubt on the quality of the training offered.

**Supplementary Information:**

The online version contains supplementary material available at 10.1186/s12992-025-01105-8.

## Introduction and background

Increasing medical doctor density has been associated with improved health system performance, reduced burden of disease, and better health outcomes such as maternal mortality ratio, under-five mortality rate, infant mortality rate, and neonatal mortality rate [1]. Notwithstanding, for the World Health Organization the world is currently experiencing “a dangerous scarcity of health workers” [2], and doctors and healthcare workers “must urgently be trained and retained” [3].

Multiple drivers have been identified for such a global scarcity of medical doctors, from the complexity of training and retention of expensive cadres in inadequately funded health systems [4], to maldistribution [5], low morale and motivation [6], and migratory pressures in an increasingly globalised healthcare labour market [7].

Modelling studies estimated that in 2019 the world had 1.67 doctors per 1,000 population worldwide, whereas a density of at least 2.07 would be needed to achieve effective coverage of UHC [8], and 3.3/1,000 in 2030 to reach the health related Sustainable Development Goals by the end of the decade [9]. A mix of recruitment and retention policies will be needed to achieve such ratios [10], but an expansion of health worker training capacity is certainly required to ensure a sufficient pool from which to recruit, particularly for those countries with the fastest growing economies and population [11].

Evaluation studies have shown that expanding national medical training in resource-scarce settings is complex, expensive, riddled with implementation challenges, and likely to bear a noticeable impact only in the longer term [12]. Scaling up government health financing has traditionally been considered the main modality for medical education expansion [13]; however, LMICs experiences in the last decade have raised concerns that rapid state expansion in medical training might be mired by implementation hurdles and put at risk the quality of education [14]. The existence of medical education markets has more recently been noticed, and private sector involvement in that market has been growing rapidly in a number of countries [4].

A substantial body of literature has focussed on the complexity of recruiting medical students from all parts of societies in LMICs [15]. In Brazil in particular, such students tend to come persistently from privileged backgrounds [16], which provided grounds for rolling-out ‘affirmative action policies’ to implement race and income quotas in the country’s public universities [17].

In 2010, the Lancet Commission on the Education of Health Professionals set out the guidelines for transforming medical education in the new century, recognising both the potential role of private financing as an additional source of investment in medical schools, but also concerns about quality and social purpose of privately owned schools [18].

In 2022 there were 3,986 operational medical schools worldwide, with a growing proportion of private colleges [19]. Although the private sector has been credited with contributing to the growth of supply [20], the rise of private for profit medical schools in lower-income countries is often viewed with suspicion, as for many, profit and educational motives are inherently antithetical [21]. On the other hand, studies from a high-income country such as Japan show that private medical schools offer a comparatively more favourable gender balance, as well as more leadership opportunities for female doctors [22].

In Brazil, the expansion of medical courses and placements has been driven by legislation and policies issued by the Federal Government in the last decade. The *Mais Médicos* (More Doctors) Law (MML) was introduced in 2013 with the stated objective to “…provide an answer to the urgent need to increase the number of doctors engaged with primary care training […] with a view to alleviate the historical scarcity of such professionals across the country” [23]. It introduced two key elements: (a) an emergency program to provide doctors in primary care to historically underserved municipalities (initially with Cuban doctors and, after 2019, primarily with Brazilian doctors); (b) the liberalisation of private undergraduate courses and student places.

While the primary care doctors measures have received most of the attention [24, 25], this article focuses on the second component of MML, and on its impact on the supply of doctors in Brazil. Since 2013, medical courses have been authorized through ad hoc legislation targeting private institutions and specific disadvantaged locations. Over the last two decades, successive Brazilian governments have implemented policies aimed at broadening access to higher education for socioeconomically underrepresented groups. This includes an increase in medical undergraduate student places in municipalities with lower physician-to-inhabitant ratios, primarily facilitated by federal universities.

The opening to private medical schools was implemented through subsequent official calls (*chamamentos públicos*) for accredited private institutions to submit proposals to open schools in selected, underserved municipalities. No public funding was made available to private schools; however, once the school/course was approved, students attending courses in such private institutions were eligible to apply for public scholarships to cover tuition fees. A parallel set of policies - The University for All Program (PROUNI) - has played a role by providing scholarships in private universities for poor students, covering different proportions of fees based on family income [26].

At the same time, public universities have gradually adjusted admission policies to enhance access for low-income, black, and indigenous students. In 2012, the *Lei de Quotas* federal law mandated that 50% of undergraduate placements in federal universities be reserved for students from public high schools, with a priority for those from low-income backgrounds, with disabilities, and self-identifying as ‘black’ or ‘indigenous’ [27]. By 2023, there were 389 medical schools in the country – up from 222 in 2013 – the majority of which (268) of private nature [28]. Preliminary studies claim Brazil’s policies for scaling up of medical training has improved access to medical training and geographical distribution of the medical workforce [29, 30].

This study asks what the impact has been of the Government of Brazil’s policies to expand the country’s medical workforce in the last twenty years, and the contribution by private medical schools. To provide an answer to such complex question, we look at four key domains, namely: the overall increase in the medical workforce, changes in the geographic distribution of doctors, changes in the socio-economic profile of the students enrolled in public and private medical schools, and the quality of teaching in those institutions.

Ultimately, our study of Brazil’s experience aims at extracting lessons on benefits and perils of using private sector policies to expand medical education in similar countries.

## Methods

Our methodological approach covers the assessment of different potential impacts of Brazil’s MML policies, namely: overall progress in doctor to population ratios; geographical location of doctors and students in disadvantaged areas; diversity of doctors and students’ population, and; quality of students’ education. We understand there will be trade-offs across such domains, as any policy is likely to notch successes as well as failures and unintended consequences in different areas, and the overall assessment will lie on the balance of such trade-offs. We ponder the limitations of such an approach in the discussion.

To assess progress in the above domains, we compare the evolution of the medical workforce and medical students in the decades before and after the 2013 policies. Our analysis focussed particularly on medical students as: (a) those policies were intended to expand medical training capacity, and; (b) such impact would be lagged and felt on the medical workforce only 5–6 years later, when medical students finished their education.

### Data sources

We employed different datasets to evaluate changes in absolute and per capita medical workforce, doctors’ geographical distribution, location and sociodemographic profile of medical students, quality of teaching of public and private medical schools (Table 1).

Data on physician workforce was extracted from the Brazilian Federal Medical Board (BFMB) registry December 2003, 2013, and 2023. Physician’s specialty information comes from the Ministry of Education and the Brazilian Medical Association, which are the two legal issuers of specialist diplomas in Brazil. Disaggregation of this data allowed the study of the spatial distribution of doctors. The Medical Demography Study Group of the Medical School of the University of São Paulo has collected and analyzed such data since 2010 [31, 32].

**Table 1:** Outcomes of interest and data sources per assessment domain

Area to be assessed	Outcomes of interest	Data sources
Changes in the medical workforce	Evolution of census of doctors over the last 20 years	Data on physician workforce was extracted from the Brazilian Federal Medical Board (BFMB).
		Physician’s specialty information comes from the Ministry of Education and the Brazilian Medical Association.
Geographical distribution of doctors	Doctors’ geographical distribution per inhabitants	Medical demography of Brazil (2000–2023)
		The population estimates for the states and municipalities were based on data from The Brazilian Institute of Geography and Statistics (IBGE).
Changes in location and composition of medical students’ population	Medical school vacancies per state	Data on medical courses and students, from 2003 to 2022, was obtained from the ‘Undergraduate Courses’ unit of the Higher Education Census, collected annually by the Anísio Teixeira National Institute for Educational Studies and Research (INEP).
	Students’ socio-demographic characteristics	
Quality of teaching	Results from students’ attainment tests	The 2019 National Student Attainment Exam (ENADE) dataset (e-MEC) was obtained from the Brazilian Ministry of Education and Culture.
	Number of staff per students	

Information on medical courses and students was obtained from the “Undergraduate courses” section of the ‘Anísio Teixeira National Institute of Educational Studies and Investigations’ database (INEP), from 2013 to 2022, based on the ‘name of the course’ according to the International Standardized Classification of Education/Unesco.

The 2019 National Student Attainment Exam (ENADE) dataset (e-MEC) was used to assess teaching quality across public and private medical schools. Such an exam is carried out in all Universities and Faculties in Brazil, and is structured into a General Education component, which assesses general skills and knowledge, and a Specific Knowledge component, which evaluates the knowledge and skills expected for the professional profile. ENADE focusses on evaluating the quality of institutions rather than individual students, is not a national ‘bar exam’, and does not prevent medical students with poor performance from obtaining their diploma and subsequent medical licence. The ENADE dataset e-MEC was obtained from the Ministry of Education and Culture [33].

### Data analysis

Aggregate student data included the number of students enrolled each year and encompasses the following characteristics: gender, self-declared race, secondary education, participation in minority reservation programs, student financing, and type of financing.

We performed descriptive statistics to examine: (a) absolute yearly increases in headcount of doctors and medical students; (b) doctors and students per 1,000 population; (c) absolute and proportional annual increase of doctor and students per type of school, and; (d) number of teaching staff per medical student, per type of school.

To analyze the increasing trend of students and physicians, interrupted time-series (ITS) analysis [34] was conducted using segmented linear regression, comparing two time periods considering the MML as the start of the intervention. Since the MML was implemented in October 2013, we considered the start of the intervention as 2014 for student numbers and 2020 for doctors’ numbers. Ordinary least-squares regression models with robust standard errors were run to estimate β-coefficients with 95% confidence intervals (CIs) to report the average annual change in the number of subjects during the preintervention period (slope of the outcome), the average number of subjects at the beginning of the intervention (compared with the counterfactual - the expected trend in the absence of the intervention, given the pre-existing trend), and the difference in the average annual change in the number of subjects between pre-intervention and post-intervention periods (slope of the outcome). Given the potential for autocorrelation in the residuals in time-series data, the Cumby-Huizinga test was run to identify whether error terms were correlated over time. Additionally, robust standard errors were used to adjust for any residual autocorrelation or heteroskedasticity, ensuring more reliable estimates of the regression coefficients and their variances [34].

Comparisons between the public and private schools ENADE quality assessment groups (ENADE 1–2, 3, and 4–5) were performed using the chi-square test, with the significance level set at 5%.

The medical demography and medical students databases were analysed through the Statistical Package for the Social Sciences (SPSS) version 26 for Windows (International Business Machines Corp, New York, USA), R-GUI version 3.5.3 [35] for statistical analysis, and Microsoft Excel for Mac version 16.8. Inequalities in the distribution of doctors were illustrated using a choropleth map, which quantitatively highlights heterogeneity through color gradients; the open source Quantum Geographic Information System software – QGIS – was used to develop the map [36].

Being based on secondary analysis of already collected data, this project was deemed exempt from ethical approval by the ethics board of the faculty of medicine of the University of São Paulo.

## Results

### Overall impact on the National workforce

Our data show there has been a consistent growth of the medical workforce in Brazil in the last twenty years, both in absolute and in per capita terms.

**Fig. 1:**
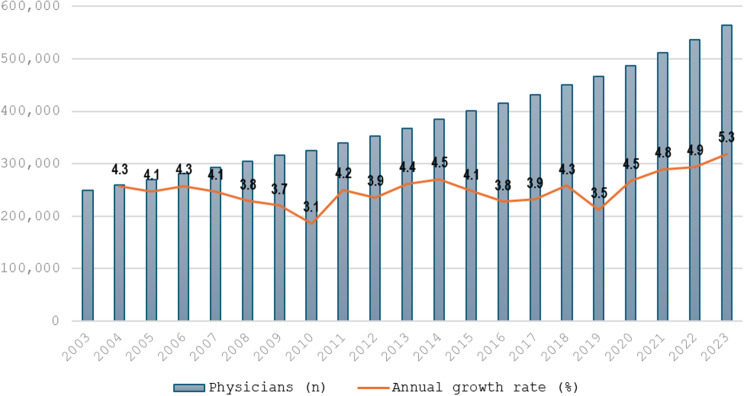
Evolution of registered medical doctors 2003–2023 and respective annual growth rate. Source: Medical Demography, University of São Paulo

Doctors’ headcount increased by 98.12%, going from 284,854 in 2003 to 564,363 in 2023. Although the workforce increased steadily each year since 2003, the largest increases were recorded after 2020; the average annual rate of increase was 4.87% for the period 2020 to 2023, as compared to a rate of 4.0% for the previous period between 2003 and 2019. Slower annual growth was particularly noticeable between 2006 and 2010 (Fig. 1).

51.9% of practicing doctors in 2023 graduated from private medical schools, up from 36.8% in 2003 [32]. Despite Brazil’s population growth of the last decade, such an increase in the medical workforce lifted the national doctor-per-population ratio from 1.42 per 1,000 population in 2003, to 1.90 in 2013, and 2.77 in 2023.

**Fig. 2:**
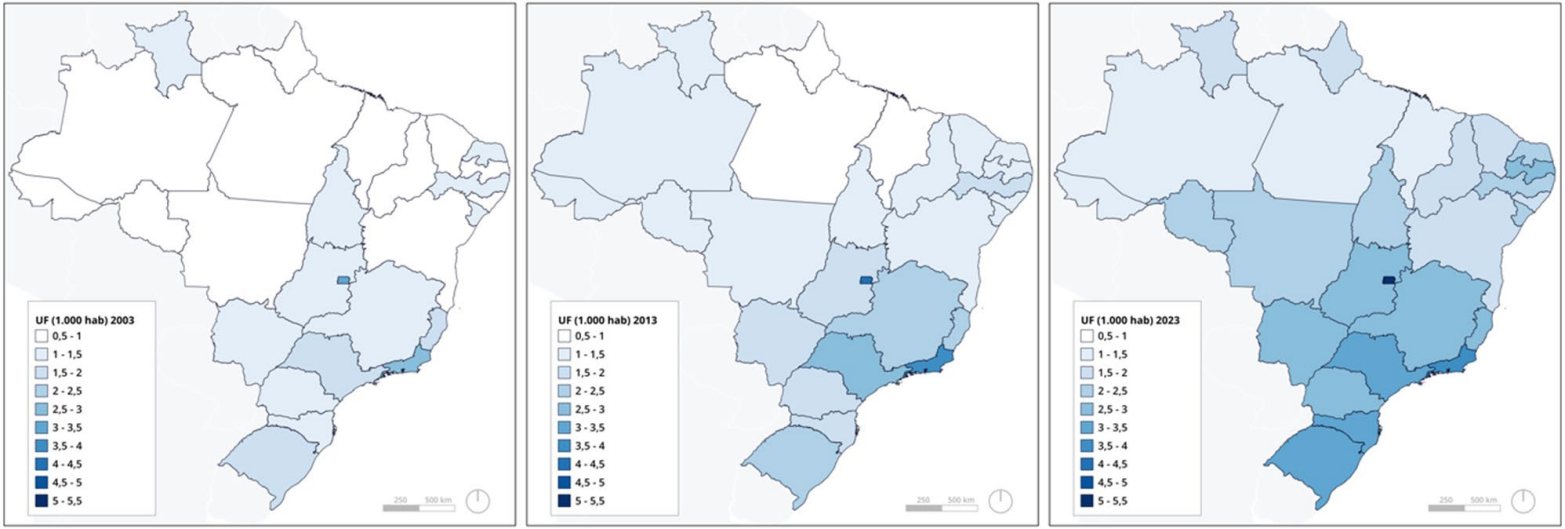
Doctors per population in Brazil’s states in 2003, 2013, and 2023. Source: Medical Demography, University of São Paulo

Each of Brazil’s 26 states saw an increase in the number of doctors per capita. Whereas in 2003 as many as 12 states had less than one doctor per 1,000 inhabitants, in 2023 all states had surpassed that threshold. Such improvement is particularly visible for northern and Amazonian states like Maranhão, Amapá and Pará that used to have fewer than 1 doctor per 1,000 inhabitants. Paraiba state in Brazil’s less affluent North-East, as well as Rio Grande do Sul and Santa Catarina from the richer South, experienced the largest absolute increases, doubling their doctor density in the last ten years (Fig. 2 below).

Our analysis at the municipality level shows that, between 2013 and 2023, doctor-per-population density improved across all types of municipalities, with less populated municipalities doubling their original doctor-to-population ratios (municipalities with less than 5,000 inhabitants went from 0.20 doctors per 1,000 in 2013 to 0.43 in 2023 - Table A1 in Annex). However, in terms of absolute numbers, it was the most populated municipalities (> 500,000) that benefitted the most from the influx of new doctors – from 3.92 in 2013 to 6.23 in 2023, equal to 61% of the overall additional supply).

### Changes in location and profile of medical students

**Fig. 3:**
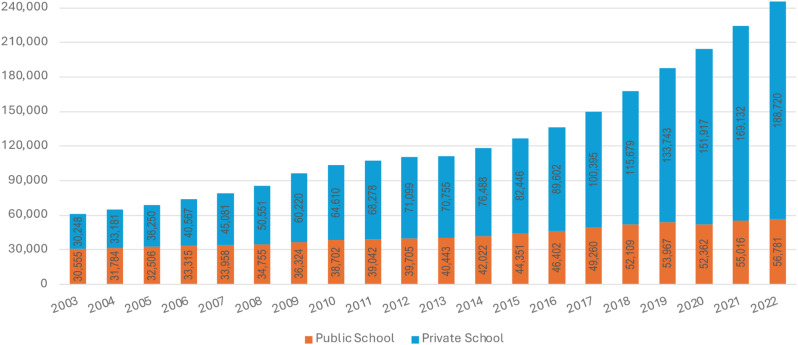
Break-down of evolution of medical students by public and private between 2003–2022. Data source: National Institute of Educational Studies and Investigations Anísio Teixeira, INEP (‘undergraduate courses’ unit of the Higher Education Census)

Our data on medical school places show there were 245,501 students enrolled in 2022 (the latest available year of consolidated data), up from 111,198 in 2013, and 60,803 in 2003. 76.87% of the 2022 students were in private schools (up from 49.75% in 2003) - Fig. 3.

The overall 2003–2022 growth was largely driven by the increase of private medical school openings after 2013, as private medical students went from 30,248 in 2003 to 70,755 in 2013, and 188,720 in 2022 – an increase of 166.72% over the last decade.

Whereas the increase in deployed doctors has been linear for the last 20 years, a substantial acceleration is visible after 2013 for medical students; their annual growth rate had reached 9.5% by 2022, while averaging 6.2% between 2003 and 2013. This followed a substantial slump in student intakes started in 2010 (Fig. A1 and A2 in annex).

Interrupted time series analysis (Table 2) showed the number of physicians increased significantly every year prior to 2020 (when new students commencing after the legislation would graduate) by 13,593 doctors (how many doctors were added per year before MML) (95%CI: 12,493.5; 14,747.7, *p* < 0.001). After the intervention, starting in 2020 there was a significant additional increase of 7,353 doctors (95%CI: 5,412.9; 9,293.5, *p* < 0.001), with evidence of an immediate change of 12,220 doctors per year after the intervention – the marginal increase above the expected trend (95%CI: 8,982.6; 15,458.2, *p* < 0.001). After the intervention, the number of doctors increased annually at a rate of 25,814 doctors (95%CI: 23,068.1; 28,560.2, *p* < 0.001).

As for medical students, prior to 2014 numbers increased every year by 5,703 (95%CI: 5,029.3; 6,377.7, *p* < 0.001). In the first year of the intervention (2014), there was a non-significant decrease in the total number of students of 13,267 students (95%CI: -27,585.2; 1,050.9, *p* = 0.067), with no evidence of an immediate change after the intervention. This was followed by a significant increase in the annual trend of students, relative to the preintervention trend, of 10,540 students per year (95%CI: 8,414.7; 12,665.5, *p* < 0.001). After the intervention, the number of students increased annually at a rate of 16,243 students (95%CI: 14,033.9; 18,453.4, *p* < 0.001).

**Table 2:** Parameter estimates of the overall effect of the more Doctors law (MML) on the annual number of students and physicians in Brazil from 2003 to 2023 (95% CI)

	Average annual change prior to onset of MML effects β1	*p*-value	Additional average annual increase after MML β2	*p*-value	Difference between average annual change before and after MML β3	*p*-value	Linear trend after the onset of the effects of the MML β1 + β3	*p*-value
Doctors (before and after 2020)*	13593.6 (12493.5; 14747.7)	< 0.001	7353.2 (5412.9; 9293.5)	< 0.001	12220.4 (8982.6; 15458.2)	< 0.001	25814.1 (23068.1; 28560.2)	< 0.001
Students (before and after 2013)*	5703.5 (5029.3; 6377.7)	< 0.001	-13267.1 (-27585.2; 1050.9)	0.067	10540.1 (8414.7; 12665.5)	< 0.001	16243.7 (14033.9–18453.4)	< 0.001

*Threshold years for the effects of MML

Our INEP data show the geographic distribution of enrolled medical students improved substantially in the 2003–2022 period. Two decades ago, 50.9% of all students were studying in large state capital city areas, whereas in 2022 only 38.7% of them were (Table A2 in annex). Comparatively, the number of medical students studying in schools based in areas with between 100,000 and 300,000 inhabitants grew from 18.8 to 23.4%, and those in areas with fewer that 100,000 went from 6.1 to 14.6% of total.

While the distribution of students across types of municipalities remained constant between 2003 and 2013, the largest increases of students in scarcely populated municipalities was recorded after 2013, particularly for those areas with fewer than 300,000 inhabitants - from 27.57 to 37.49% in the last decade (Table A2 in annex). Most of the students in 2022 (62.5%) were still based in schools in municipalities with > 300,000 inhabitants, but such proportion was smaller than ten years earlier (72.4%).

In terms of their socio-demographic profile, there are suggestions that diversity has increased over the last decade. Self-declared ‘White’ students still represent 68.7% of the 2022 cohort (it was 71.6% in 2013 although the majority of students failed to declare their race^1^). A substantial increase of self-declared ‘*Pardos’* (mixed-race) is noticeable in 2022–25.5%, up from 19.4%. All the other minority groups grew more than the average rate of increase of the overall student population (121%) (Table 3).

**Table 3:** Evolution of students’ socio-demographic profile in public and private medical schools

Student SEC	All medical school				Public medical school				Private medical school			
	2013		2022		2013		2022		2013		2022	
	*N*	%	*n*	%	*n*	%	*n*	%	*n*	%	*n*	%
**Self-reported race**												
‘White’ (Caucasian)	37,786	71.6	148,861	68.7	12,580	59.8	27,259	51.8	25,206	79.4	121,602	74.1
‘Black’ (African origin)	3012	5.7	7535	3.5	2450	11.6	3877	7.4	562	1.8	3658	2.2
‘Pardos’ (mixed race)	10,233	19.4	55,361	25.5	5184	24.6	19,683	37.4	5049	15.9	35,678	21.7
‘Yellow’ (Asian origin)	1597	3.0	4127	1.9	701	3.3	1175	2.2	896	2.8	2952	1.8
‘Indigenous’	164	0.3	900	0.4	117	0.6	586	1.1	47	0.1	314	0.2
Total	52,792	100.0	216,784	100.0	21,032	100.0	52,580	100.0	31,760	100.0	164,204	100.0
**Gender**												
Male	49,506	44.5	94,711	38.6	20,163	49.9	28,608	50.4	29,343	41.5	66,103	35.0
Female	61,692	55.5	150,790	61.4	20,280	50.1	28,173	49.6	41,412	58.5	122,617	65.0
Total	111,198	100.0	245,501	100.0	40,443	100.0	56,781	100.0	70,755	100.0	188,720	100.0
**Secondary education**												
Public	19,293	24.2	78,374	31.9	10,127	35.2	29,024	51.2	9166	17.9	49,350	26.2
Private	60,525	75.8	167,070	68.1	18,616	64.8	27,709	48.8	41,909	82.1	139,361	73.8
Total	79,818	100.0	245,444	100.0	28,743	100.0	56,733	100.0	51,075	100.0	188,711	100.0
**Minority reservation places**												
Yes	5169	4.6	22,145	9.0	5063	12.5	21,557	38.0	106	0.1	588	0.3
No	106,029	95.4	223,356	91.0	35,380	87.5	35,224	62.0	70,649	99.9	188,132	99.7
**Total**	111,198	100.0	245,501	100.0	40,443	100.0	56,781	100.0	70,755	100.0	188,720	100.0

In the 2022 cohort, female students were 61.4%, compared to 55.5% in 2013, while the proportion of students from state secondary schools was 31.9%, compared to 24.2% ten years earlier.

When comparing students’ profiles across private and public medical schools in 2022, private schools reported more self-declared ‘White’ (74.1% Vs 51.8%), and Female (65.0% Vs 49.6%) students, predominantly from private secondary school backgrounds (73.8% Vs 48.8%) (Table 3). Our data show progress in relation to ethnic diversity has been quite modest across the board; overall, the proportion of those who declared themselves ‘White’ fell from 71.6 to 68.7% with a larger reduction in public schools (59.8–51.8%) than in private schools (79.4–74.1%). The proportion of ‘Minority Reservation Places’ programme (income and race minority quotas) among public medical schools was 38% in 2022 (up from 12.5% ten years earlier), but only 0.3% for private medical school students.

### Quality of public and private medical schools

**Fig. 4:**
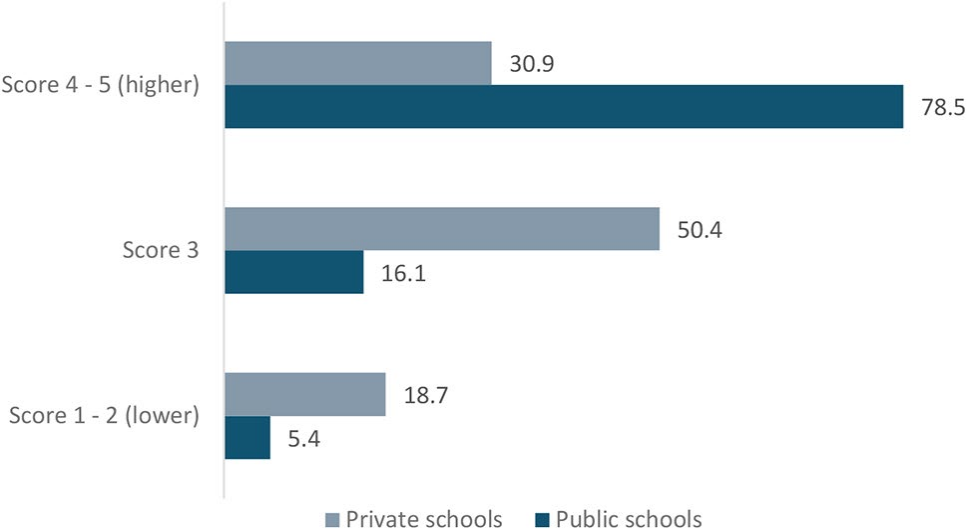
ENADE test scores by type of medical school (2019) Source: e-MEC. Score results for medical schools (93 public and 139 private)

In terms of quality of teaching, in the 2019 national students’ attainment (ENADE) evaluation tests, 78.5% of public schools achieved top average scores (level 4 and 5), compared with 30.9% of private ones (*p* < 0.001). At the same evaluation, as many as 18.7% of private schools received the lowest scores (levels 1 and 2), compared to 5.4% of public schools (*p* < 0.001) (Fig. 4).

As per class size and tutor qualifications, 38.9% of students in public medical schools were taught in classes of more than 100 individuals, whereas the corresponding proportion was 80.4% for private medical schools (Figure A3 in annex).

The student-to-faculty-member ratio in private medical schools in 2022 was considerably larger than in public ones (7.5 Vs 3.9). A smaller proportion of the teaching staff in private medical schools had a doctorate qualification, as the ratio of students to a PhD faculty member was over three times larger in private schools than in public ones.

## Discussion

Our analysis indicates that Brazil’s MML succeeded in expanding the country’s medical workforce at a rate higher than would otherwise have occurred without its policies. Although the growth had started before 2013 [30], our medical demography evidence suggests that the MML policies accelerated the rate at which medical students are trained and incorporated into the workforce. The student data are most convincing on this point, and the analysis suggests they began to increase about a year after the legislation, which is a credible response lag.

MML policies also regulated the growth that took place after 2013 to produce qualitative changes in geographical distribution and student diversity; however, results in this respect have been more mixed. Although progress has been made, medical students in Brazil are still predominantly white from affluent backgrounds; even though a growing proportion of students have been trained in underprivileged states and all municipalities now have at least one doctor per 1000 population, we show it is still the richer urban municipalities that have benefited the most from the increased supply of doctors. Evidence from other studies aligns with this finding; the country-wide benefits of MML seem to have been undermined by the widespread attraction of doctors to non-priority areas and local substitution effects [24], and in São Paulo state, recently graduated primary care doctors are increasingly opting for short(er) tenure in urban hospitals in place of the rural primary care roles envisaged [37].

The data on students’ attainment and student-to-staff ratios suggest that quality of education in private medical schools in Brazil is lower than in public ones. This finding is consistent with some other literature on this topic from other large, economically emerging middle-income nations [38, 39], and raises concerns about the suitability of private colleges as a tool to rapidly expand health workforces where regulatory capacity is limited. Since alternative public-only approaches have not produced better results, continuing to support a mix of public and private sector policies while strengthening regulation, might represent the most promising strategy for the expansion of health workforces, despite the concerns above [40]. Regulations need to better address the definition, implementation, and monitoring of quality standards of private medical schools, to steward the private sector towards public objectives, albeit the hard constraints of resources (both financial and human) that regulation cannot address [41].

Our analysis suggests that private schools have contributed only marginally to improve diversity in Brazil’s medical workforce, as the gains recorded in this area are more likely to be attributable to the quotas applied in public universities. Given the large tuition fees, it is hardly surprising that private schools mostly attract students from privileged backgrounds [42]. On the other hand, MML’s minority quotas implemented in Brazil’s public universities appear to have had more impact. If confirmed, we believe our findings make an important contribution to the current debate on affirmative action, racial diversity, and access to higher education in Brazil [43] and elsewhere to the extent that the required private investment is open to only more privileged social groups everywhere [44].

Brazil is an upper middle-income country which experienced rapid economic growth between 2003 and 2013 (with GDP growing by nearly 50% over this decade) and much slower growth between 2013 and 2023 (with total GDP growing by less than 10% over the decade) [45]. This is important context for assessing the economic conditions of other similar countries in which the market alone might be likely to drive an expansion of the medical workforce, and under which supportive policies might be influential.

Other contextual factors are likely also to be important for governments planning to scale-up health workforces. Expanding medical school places will only result in expanding student numbers if there is pent up demand for medical education among high school graduates with adequate attainment levels to be able to cope with medical education [46]. Given the increased capacity in the private sector, that demand needs to be backed by willingness and ability to pay fees that have been set at roughly double the average per capita income in Brazil or other middle-income countries.

The outcomes of the increasing numbers of medical graduates for the geographical distribution of medical workforce are also contingent on labour market conditions in situ. A number of those conditions determine whether new graduates will fill vacancies in primary care in more remote locations [47]. Evidence that returns are insufficient to motivate accepting roles in such locations from São Paolo State [37] is suggestive of what is probably the national situation. Improving terms and conditions for primary care posts must become a policy objective for them to become the preferred choice for a sufficient number of graduates. This is likely to be a consideration in most countries, given the relevance of the Brazilian experience.

Going forward, regulatory measures should be considered to mitigate the potential negative impacts of the rapid increase in the number of physicians in Brazil [48], with the lessons learned serving as a model for other countries seeking to expand the supply of healthcare professionals. The quality of physicians will depend on monitoring and assessing quality and diversity of the large cohort of doctors from newly established medical schools [49]. National scaling up of the medical workforce driven primarily by the growth of private medical schools should be accompanied by regulations ensuring the social inclusion of students, weighting for entry scores to better balance diversity in medical admission systems [50], and curricular guidelines aimed at prioritizing training for practice within the public healthcare system.

Brazil’s bold experiment offer a source of some optimism for governments aiming at scaling-up medical workforces. Firstly, it shows how a combination of public and private sector policies can be harnessed for a rapid expansion of the workforce, as long as there is a large enough national pool of quality students, unmet demand for medical education, and ability to pay, which is likely to be the case for many emerging economies [51].

Although the private sector can be harnessed to rapidly increase the medical workforce, close monitoring of private schools’ outputs must accompany such development, as private schools seem to do little to address equity and diversity of the future doctors they train, and the quality of the training offered appears to be lower than in public schools [49]. Therefore, concomitant public sector policies could be considered to support diversity of student populations and keep high standards of medical training, for example following a model of market regulation by participation of public enterprises [52].

Once trained, new graduates will inevitably feel a pressure to relocate to more profitable geographical areas as a way of recouping the investment made in their education [4]. Public sector policies will be needed to offset earning opportunities differentials between urban and rural posts, and make the latter an attractive choice of location [53].

We acknowledge a few limitations in our analysis. First, the *Mais Médicos* Legislation is not a single homogeneous policy, but rather a sequence of diverse supply-side interventions implemented since 2013 and spanning across multiple years. As a result, it was difficult to pin-point the specific effect of such measures on the market for physicians. Secondly, despite our team’s decade-long effort to collate and consolidate physician data [32, 49, 54], some socio-demographic data and school enrolment data are still missing for physicians and medical students, particularly for older datasets. In the 2013 dataset in particular, there were missing data for some our socio-demographic characteristics of interest, particularly ‘race/colour’ and ‘type of secondary education (public or private)’, primarily due to underreporting during the earlier years of data collection, as these variables only started being systematically collected in later years (e.g., race/color in 2007). We recognise this has left some of our analysis incomplete. Finally, the power of our interrupted time series analysis for doctors may have been affected by the short time considered after the intervention (three years), and by possible confounders affecting the supply of doctors over the last twenty years [34].

## Conclusions

Expanding national medical workforces to increase population access to health services is a global preoccupation for governments in resources-constrained settings. However, few examples exist of successful public or private sector policies to carry out such scaling-up. We used evidence from original medical demography and medical students’ datasets from Brazil to analyse the supply of doctors in the decades before and after the introduction of its *Mais Médicos Legislation*, a set of policies aimed at liberalising the medical education market and implementing quotas to improve its supply of physicians.

Our study suggests that Brazil’s policy approach has delivered a substantial overhaul of its medical workforce through a combination of public and private sector interventions, and that there might be scope to address workforce shortages using strategies similar to those that have been employed in Brazil. Our analysis shows that private medical schools might be relied upon for rapid expansion of medical workforces, as long as the quality of their teaching and diversity of students is closely monitored, and public schools pursue the objective of balancing the national workforce.

These seem to be in fact important caveats with respect to the quality and diversity implications of such heavy reliance on private initiative to expand training opportunities. All countries will have to consider how those caveats apply in context, and the trade-offs involved between quality, geographical distribution and equal opportunities on the one side, and numbers and availability on the other.

## Electronic supplementary material

Below is the link to the electronic supplementary material.

Supplementary Material 1

## Data Availability

No datasets were generated or analysed during the current study.

## Footnotes

1 In 2013, 52.5% of students failed to declare their race, while in 2022 only 11.7% did.
