# Supplementary material for: Brazil’s experiment to expand its medical workforce through private and public schools: Impacts and consequences of the balance of regulatory and market forces in resource-scarce settings
Source: Global Health. 2025 Mar 28;21:14. doi: 10.1186/s12992-025-01105-8 (PMC11951509; doi:10.1186/s12992-025-01105-8)
Supplement: Supplementary file 1 — Supplementary Material 1 [file 12992_2025_1105_MOESM1_ESM.docx]

# STATISTICAL ANNEX

Table A1: Doctors density per type of municipalities

| **Type of municipality per population** | **2013** | | **2023** | |
| --- | --- | --- | --- | --- |
|  | **N** | **Density** | **N** | **Density** |
| ≥ 500 mil | 232,312 | 3.92 | 366,737 | 6.23 |
| 100 a 500 mil | 94,256 | 1.79 | 142,437 | 2.51 |
| 50 a 100 mil | 22,831 | 0.97 | 39,205 | 1.67 |
| 20 a 50 mil | 18,915 | 0.58 | 31,652 | 0.99 |
| 10 a 20 mil | 6,415 | 0.33 | 10,801 | 0.56 |
| 5 a 10 mil | 2,202 | 0.25 | 4,063 | 0.49 |
| ≤ 5 mil | 856 | 0.20 | 1,901 | 0.43 |

Table A2: Evolving geographic distribution of medical students 2003-2022, selected years

| **Year** | **Medical Students in Densely and Scarcely Populated Municipalities** | | | | | | | |
| --- | --- | --- | --- | --- | --- | --- | --- | --- |
|  | State capital city areas | | Non-capital areas with >300,000 inhabitants | | Areas with 100,000 to 300,000 inhabitants | | Areas with fewer than <100,000 inhabitants | |
| 2003 | 30,716 | 50.50% | 14,704 | 24.18% | 11,444 | 18.82% | 3,698 | 6.08% |
| 2006 | 36,982 | 50.05% | 16,839 | 22.79% | 14,131 | 19.12% | 5,930 | 8.02% |
| 2010 | 52,977 | 51.27% | 20,651 | 19.98% | 20,382 | 19.73% | 9,302 | 9.00% |
| 2013 | 54,759 | 49.24% | 25,767 | 23.17% | 21,072 | 18.94% | 9,600 | 8.63% |
| 2016 | 63,173 | 46.44% | 32,442 | 23.85% | 27,180 | 19.98% | 13,209 | 9.71% |
| 2019 | 78,519 | 41.82% | 45,772 | 24.38% | 42,123 | 22.44% | 21,296 | 11.34% |
| 2022 | 94,918 | 38.66% | 58,507 | 23.83% | 57,541 | 23.43% | 34,535 | 14.06% |
|  | | |  |  |  |  |  |  |

Data source: National Institute of Educational Studies and Investigations Anísio Teixeira, INEP (‘undergraduate courses’ unit of the Higher Education Census). Population estimates from 2013 to 2022 were based on The Brazilian Institute of Geography and Statistics (IBGE)

Figure A1: Medical students’ headcount and annual growth rate 2003-2023


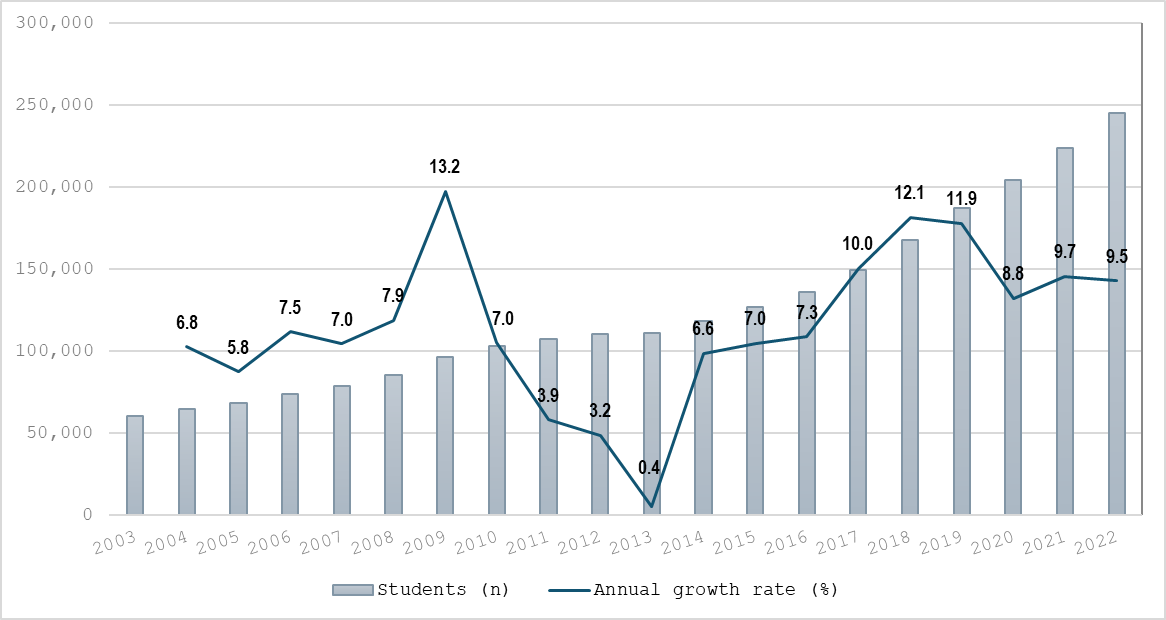


Figure A2: Annual growth rates for registered doctors and medical students, 2003-2023

Figure A3: Proportion of medical students type of classes and student to teacher ratios, by type of medical school (2022)>
